# Risk factors of positive depression screening during the third trimester of pregnancy in a Chinese tertiary hospital: a cross-sectional study

**DOI:** 10.1186/s12888-023-05343-1

**Published:** 2023-11-09

**Authors:** Ying Sun, Xiaobo He, Xuejun Gu, Xiuping Yang

**Affiliations:** 1 Department of Women Health Care, Ningbo Women and Children’s Hospital, Ningbo, Zhejiang China; 2 Department of Obstetrics, Ningbo Women and Children’s Hospital, Ningbo, Zhejiang China

**Keywords:** Depression, Depressive symptom, Edinburgh postnatal depression scale, Influencing factors, Late pregnancy

## Abstract

**Objective:**

Pregnant women experience enormous psychological pressure, particularly during the late trimester. Symptoms of depression in late pregnancy may persist postpartum, increasing the incidence of postpartum depression. This study is aimed to investigate the factors influencing depressive symptoms among pregnant women in their third trimester at a Chinese tertiary hospital and provide information for effective intervention.

**Methods:**

Pregnant women in their third trimester who visited the Ningbo Women and Children’s Hospital between January 1, 2020 and June 30, 2022 participated in this study. A score of ≥ 13 on the Edinburgh Postnatal Depression Scale (EPDS) was considered as positive for depressive symptom. Potential influencing factors were examined by using an online questionnaire and analyzed using multivariate logistic regression.

**Results:**

A total of 1196 participants were recruited. The mean EPDS score was 7.12 ± 4.22. The positive screening rate for depressive symptom was 9.9%. Univariate analysis showed that living with partner, annual family income, planned pregnancy, sleep quality, and partner’s drinking habits were related to positive screening for depression(*P* < 0.05). Furthermore, multivariate logistic regression analysis showed that living away from the partner (odds ratio [OR]: 2.054, 95% confidence interval [CI]: 1.094–3.696,* P* = 0.02), annual family income < 150,000 Chinese Yuan (CNY; OR: 1.762, 95% CI: 1.170–2.678,* P* = 0.007), poor sleep quality (OR: 4.123, 95% CI: 2.764–6.163,* P* < 0.001), and partner’s frequent drinking habit (OR: 2.227, 95% CI: 1.129–4.323,* P* = 0.019) were independent influencing factors for positive depression screening (*P* < 0.05).

**Conclusion:**

Family’s economic condition, sleep quality, living with partner, and partner's drinking habits were related to positive depression screening in late pregnancy. Pregnant women with these risk factors should be given more attention and supported to avoid developing depression.

## Introduction

Pregnancy is a period of significant change in a woman's physiology and psychology [1]. During pregnancy, women are vulnerable to the negative impacts of life events that may cause depressive symptoms. The prevalence of prenatal depressive symptoms is approximately 14%-23%, and is a major global public health concern [2–4]. Prenatal depression may reduce self-care ability, cause an increase in maternal and infant incidences such as poor pregnancy outcomes [4], higher postpartum depression, and negative impacts on fetal and child health [2, 5]. The third trimester is the link between preconception and postnatal needs and is an important node for women to become mothers. A recent study found that depression inlate pregnancy and postpartum period was strongly correlated (*r* = 0.706, *P* < 0.001) [6]. In the third trimester, pregnant women experience more physical changes, greater psychological burden, and an increased probability of pregnancy complications, which increase the risk of depressive symptoms.

There are several studies about the influencing factors for depressive symptoms in western countries [7–11]. It is reported that factors related to depressive symptoms in pregnant women included history of depression, negative obstetric experiences, lack of social support, low education, and alcohol consumption and smoking. However the results between these factors and pregnancy-induced depression are not completely consistent. For socio-demographic factors such as age, for example, some studies found an association with younger maternal age [12], some studies have found they opposite [13, 14], while some studies have also found that there is no significant correlation between age and depressive symptoms [15, 16]. In terms of educational attainment, many studies have found that pregnant women with lower levels of education are more likely to suffer from depression during pregnancy [17–20]. Another studie [21] showed that the longer the number of years of schooling, the higher the risk of depression, while some studies reported no associations between years of schooling and depression [16, 22, 23]. Pregnancy characteristics such as planned pregnancy [7], history of abortion [8] and Psychosocial factors such as socioeconomic status, studies have found that socioeconomic factors are closely associated with the risk of depressive symptoms in pregnant women [24, 25]. However, some previous studies did not observe this association [26, 27]. Different regions have different influencing factors. So far, depressive symptoms were rarely reported in China. Ningbo is a port city with a highly mobile population, with migrant pregnant women from all over the country accounting for nearly half of all pregnant women, while none studies focused on depression in pregnancy up to now. Therefore, the aim of this study was to investigate the factors influencing depressive symptoms among pregnant women in late pregnancy and to provide evidence for effective intervention.

## Materials and methods

### Study design and participants

This cross-sectional study was approved by the Medical Ethics Committee of Ningbo Women and Children's Hospital (EC2022-045) and included in the cohort study of “Study for the epidemiology of perinatal depression in China”, which was registered in Chinese Clinical Trial Registry (ChiCTR1900027020). Pregnant women in the third trimester (28–42 gestational weeks) who visited Ningbo Women and Children's Hospital from January 1, 2020, to June 30, 2022, were recruited as the participants. Informed consents were obtained from all participants.

Inclusion criteria included: pregnant women in late pregnancy lived for more than 6 months in Ningbo City and had signed informed consent (gestational week: 28 weeks and beyond).

Exclusion criteria included: (1)patients with mental or psychological disorders before pregnancy; (2)patients with hypertension, diabetes, malignancies, chronic anemia, heart diseases, liver diseases, and kidney diseases before pregnancy;(3) those who were unable to complete the questionnaire due to communication impairment or reading disorders.

### Sample size

The required sample size was calculated using the formula**:**

$$\mathrm{n }= {\left(\frac{{Z}_{1} - \alpha /2}{\delta }\right)}^{2} \times p \times \left(1 -p\right)$$

Where, $\mathrm{\alpha }=0.05$, ${\mathrm{z}}_{1-\alpha /2}=1.96$, $p=50\mathrm{\%}$(as the positive depression screening rate was unknown, *p* was set at 50% to estimate the largest sample size), allowable error $\updelta =0.03$. The sample size was calculated as 1067 at 95% confidence limit. With a 15% of non-response rate, $\mathrm{N}=1067\div \left(1-15\%\right)=1255$.

### Depressive symptom assessment

Following the “Expert Consensus on Maternal Mental Health Management (2019)” [28], we used the Chinese version of the Edinburgh Postnatal Depression Scale (EPDS) to assess depression. Chinese version of the EPDS, which has been tested in Hong Kong [29] and Beijing [30] and has showen good reliability and effectiveness. The scale can be completed in approximately 5 min and the total score is calculated by adding item scores. Responses to the questions are scored from 0–3 based on the problem severity; question numbers 3 and 5–10 are reverse scored. Higher scores indicate more depressive symptoms. Babu Ram et alrevealed that a cutoff score of 13 yields a sensitivity and specificity of 92% and 95.6% among Asian women, respectively [31]. Therefore, cut-off score of a total score ≥ 13 points or one score ≥ 1 for item 10 was considered as positive depression screening in this study.Those with positive results were advised to undergo a comprehensive clinical interview for further evaluation, and appropriate treatment was provided. Non-pharmacological and pharmacological therapies were used for the patients.

### Exposure variables

Exposure variables were measured using an online questionnaire. Participants responded tothe online questionnaire after understanding the quick response (QR) codes and signed an informed consent.

The exposure variables were: (1) basic parameters, (2) pregnancy and delivery information, (3) lifestyle, (4) partnerinformation (Table 1).

**Table 1:** Basic, pregnancy, lifestyle, and spouse characteristics of the patients in positive and negative depression screening groups

Variables	Sample Number	Positive screening (*n* = 119, %)	Negative screening (*n* = 1077, %)	χ2	*P* value
**Basic information**					
Age				0.159	0.691
< 20	8	1(12.5)	7(87.5)		
–35	1035	104(10.0)	931(90.0)		
≥ 35	153	14(9.2)	139(90.8)		
Progestation BMI (kg/m^2^)				0.169	0.681
< 24	983	96(9.8)	887(90.2)		
≥ 24	213	23(10.8)	190(89.2)		
Marital status				0.010	0.919
Married	1159	115(9.9)	1044(90.1)		
Unmarried or divorced	37	4(10.8)	33(89.2)		
Only child or not				3.292	0.070
Yes	390	30(7.7)	360(92.3)		
No	806	89(11.0)	717(89.0)		
Family history of psychosis				0.435	0.509
Yes	17	3(17.6)	14(82.4)		
No	1179	116(9.8)	1063(90.2)		
Education level				4.104	0.251
Junior high school or lower	171	23(13.5)	148(86.5)		
High school, technical secondary school	153	18(11.8)	135(88.2)		
Junior college and bachelor’s degree	806	71(8.8)	735(91.2)		
Master's degree or higher	66	7(10.6)	59(89.4)		
Occupation				4.279	0.118
Full-time	710	59(8.3)	651(91.7)		
Freelancer	264	33(12.5)	231(87.5)		
Unemployed or others	222	27(12.2)	195(87.8)		
Family annual income				9.673	0.002
< 150,000CNY	592	75(12.7)	517(87.3)		
≥ 150,000CNY	604	44(7.3)	560(92.7)		
**Pregnancy and delivery information**					
Primipara	753	72(9.6)	681(90.4)	0.342	0.559
Abortion history	447	36(8.1)	411(91.9)	2.864	0.091
Planned pregnancy	808	67(8.3)	741(91.7)	7.639	0.006
Natural conception	1089	114(10.5)	975(89.5)	3.652	0.056
Single birth	1137	112(9.9)	1025(90.1)	0.254	0.614
Pregnancy comorbidities / complications	707	70(9.9)	637(90.1)	0.005	0.946
**Pregnancy comorbidities / complications in details**					
Threatened abortion	295	29(9.8)	266(90.2)	0.006	0.937
Gestational diabetes mellitus	243	19(7.8)	224(92.2)	1.546	0.214
Hypertension during pregnancy	41	4(9.8)	37(90.2)	0.000	1.000
Preeclampsia	13	2(15.4)	11(84.6)	0.037	0.847
Intrahepatic cholestasis of pregnancy	18	3(16.7)	15(83.3)	0.316	0.574
Abnormal fetal position	122	17(13.9)	105(86.1)	2.407	0.121
Abnormal placental location	99	11(11.1)	88(88.9)	0.162	0.678
Abnormal amniotic fluid	213	27(12.7)	186(87.3)	2.150	0.143
Abnormal fetal umbilical blood flow	63	8(12.7)	55(87.3)	0.561	0.454
Fetal intrauterine growth restriction	32	4(12.5)	28(87.5)	0.036	0.850
**Lifestyle information**					
Sports				0.164	0.686
Yes	442	46 (10.4)	396(89.6)		
No	754	73 (9.7)	681(90.3)		
Sleep quality				50.020	< 0.001
Good-sleep	915	60 (6.6)	855(93.4)		
Poor-sleep	281	59 (21.0)	222(79.0)		
Drinking before pregnancy				0.544	0.461
Never	732	69 (9.4)	663(90.6)		
Occasionally	463	50 (10.8)	413(89.2)		
Frequently	1	0 (0.0)	1(100.0)		
Drinking during pregnancy				0.057	0.812
Never	1180	117 (9.9)	1063(90.1)		
Occasionally	15	2 (13.3)	13(86.7)		
Frequently	1	0 (0.0)	1(100.0)		
Smoking during pregnancy				0.373	0.541
Never	1148	112 (9.8)	1036(90.2)		
< 100 cigarettes	33	6 (18.2)	27(81.8)		
≥ 100 cigarettes	15	1 (6.7)	14(93.3)		
Live away from spouse	94	17(18.1)	77(81.9)	7.536	0.006
Live with parents	208	19(9.1)	189(90.9)	0.187	0.666
Live with parents-in-law	336	34(10.1)	302(89.9)	0.015	0.903
Live with compatriots	63	9(14.3)	54(85.7)	1.395	0.238
**Spouse information**					
BMI (kg/m2)				0.001	0.975
< 24	675	67 (9.9)	608(90.1)		
≥ 24	521	52 (10.0)	469(90.0)		
Education level				4.175	0.243
Junior high school or lower	168	23 (13.7)	145(86.3)		
High school, technical secondary school	224	25 (11.2)	199(88.8)		
Junior college or bachelor's degree	741	66(8.9)	675(91.1)		
Master's degree or higher	63	5 (7.9)	58(92.1)		
Drinking				11.692	0.003
Never	297	28 (9.4)	269(90.6)		
occasionally	804	72 (9.0)	732(91.0)		
Frequently	95	19 (20.0)	76(80.0)		
Smoking during pregnancy				1.941	0.379
Never	574	50 (8.7)	524(91.3)		
< 100	272	31 (11.4)	241(88.6)		
≥ 100	350	38 (10.9)	312(89.1)		

The signifance of bold font is ‘*P* < 0.05’, and the results are statistically significant

*BMI* body mass index

Body mass index (BMI) “before pregnancy” was defined as the value for the “latest 3 months before pregnancy.” Annual family income was defined as the total income of the participant and her partner in the past year, including salary, financial products, and rental income, etc.Sports (Yes) impliedexercising except for walking three times per week and 30 min per session. Drinking conditions were categorized into never, occasionally, and frequently; drinking once weekly was defined as frequent drinking. The smoking status was categorized into ≥ 100 cigarettes during the whole pregnant period, < 100 cigarettes during the whole pregnant period, and never by far. Living separatey from the partner for more than 3 months per year was defined as living away from the partner.

Specially trained nurses helpedthe participants understand the questionnaire content accurately.

### Statistical analysis

GraphPad Prism 9.0 (GraphPad Software, La Jolla, CA, USA) was used to process the data. The counting data were expressed in frequency (percentage). Differences between groups were compared with χ^2^ inspection, and variables with *P* < 0.05 were included in the multivariate logistic regression analysis. *P* < 0.05 was considered as statistically significant.

## Results

### General information

**Fig. 1:**
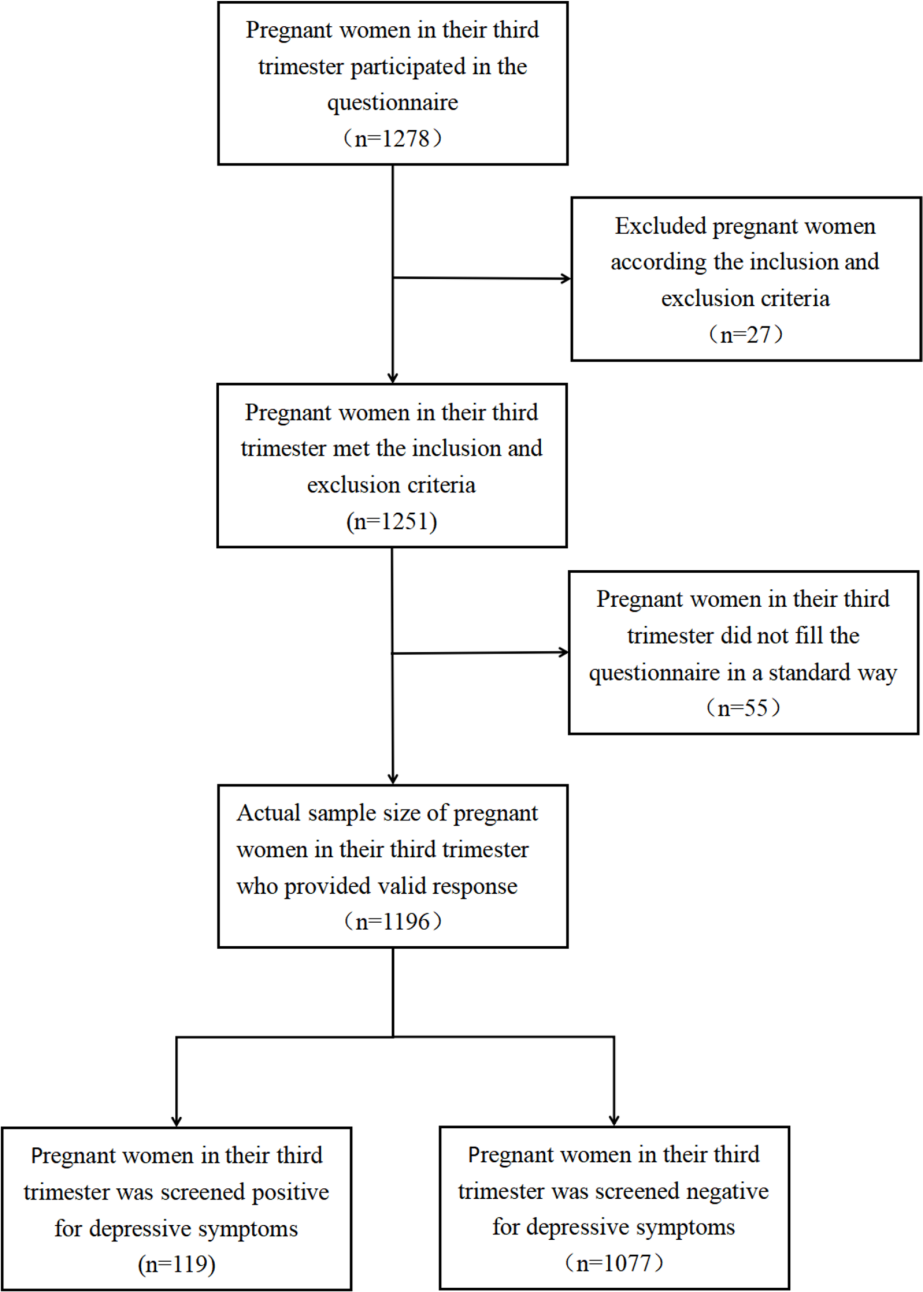
Flow diagram of patient selection

In this study, 1278 participants responded to the questionnaire, and 1196 (93.58%) provided valid response (Fig. 1). The remaining were excluded due to incompleteness of responses and withdrawal of participation. The participants' average ages were 29.57 years(SD: 4.35), average gestational weeks were 37.16(SD:2.89), and local residents comprised 40.65%(486/1196). Primiparas accounted for 62.96% (753/1196) and the restwere multiparas. Singleton and twin pregnancy comprised 95.07% (1137/1196) and 4.93% (59/1196), respectively. Among the participants, 119 (9.9%) were screened positive for depressive symptoms. The mean EPDS score was 7.12 (SD:4.22).

### Univariate analysis

Annual family income, planned pregnancy, sleep quality, living away from partner, and partner's drinking habit were significantly affected depression screening (*P* < 0.05) (Table 1).

### Multivariate logistic regression analysis

**Fig. 2:**
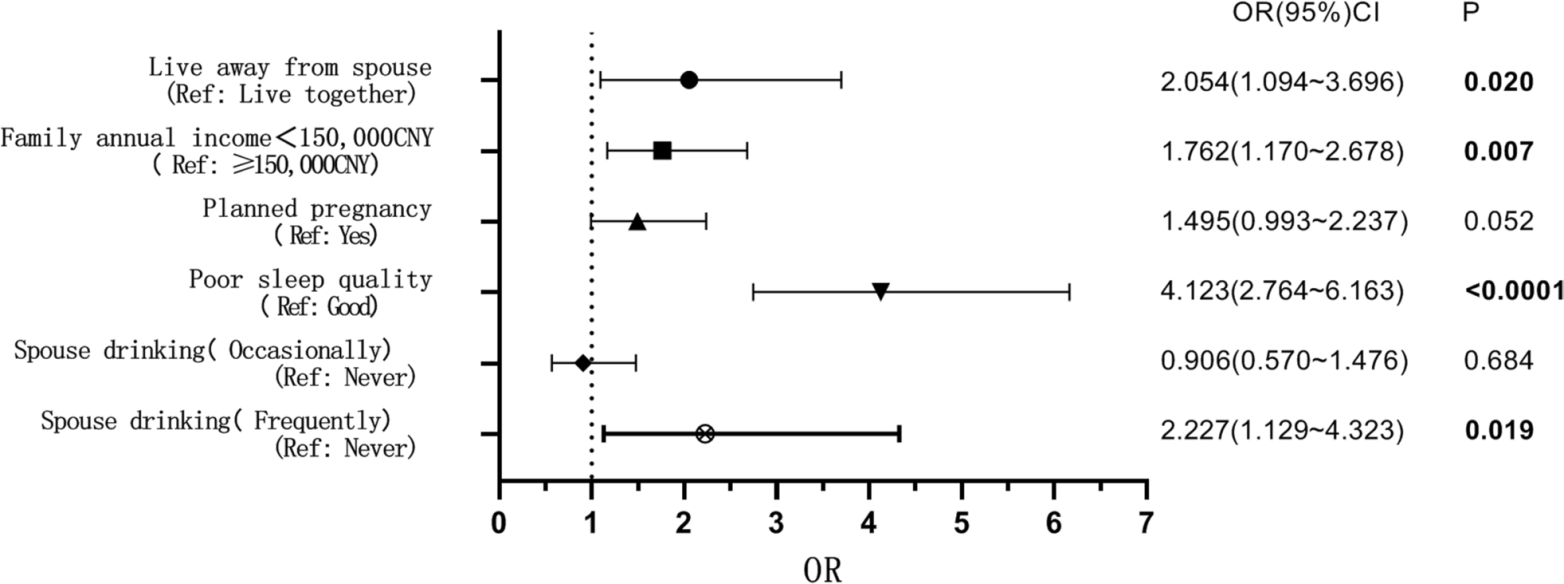
Logistic analysis of positive late pregnancy depression screening

Table 2 and Fig. 2 showed that living away from partner(odds ratio [OR]: 2.054, 95% confidence interval [CI]: 1.094–3.696, *P* = 0.02), annual family income < 150,000CNY(OR: 1.762, 95% CI: 1.170–2.678, *P* = 0.007), poor sleep quality(OR: 4.123, 95% CI: 2.764–6.163, *P* < 0.001), and partner’s frequent drinking habit (OR: 2.227, 95% CI: 1.129–4.323, *P* = 0.019) were independent influencing factors of screening positive for depression (*P* < 0.05).

**Table 2:** Logistic analysis of positive late pregnancy depression screening

	OR	95% CI	P
Live away from spouse(yes vs. no)	2.054	1.094–3.696	0.020
Family annual income < 150,000CNY (yes vs. no)	1.762	1.170–2.678	0.007
Planned pregnancy(no vs. yes)	1.495	0.993–2.237	0.052
Sleep quality(bad vs. fine)	4.123	2.764–6.163	< 0.0001
Spouse drinking( occasionally vs. never)	0.906	0.570–1.476	0.684
Spouse drinking( frequently vs. never)	2.227	1.129–4.323	0.019

## Discussion

In this study, 1196 (93.58%) valid questionnaires were obtained. The mean EPDS score was 7.12, and 9.9% of the participants were screened positive for depressive symptoms. Living away from partner, low annual family income, poor sleep quality, and partner’s frequent drinking habit were independent influencing factors of late pregnancy depressive symptoms. In this study, the mean EPDS score was 7.12 (SD:4.22), which was higher than some European countries such as Spain 5.73 [32], Hungary 5.36 [33], Sweden 5.75 [34]. China is a developing country with lower household incomes, lower levels of health care than Europe countries, and a lack of attention to mental health care, which can lead to high scores. This difference in scores needs to be confirmed by high-quality systematic reviews and another Subscale analysis of EPDS (anhedonia, depression anxiety) can also be used in future [35].

Studies from various countries showed a prevalence rate of screened positive for depressive symptoms was 8.1%–46.8% [36–38]. In our study, the rate (9.9%) was lower than that in other developing countries such as India (35.7%) [39], Thailand (46.8%) [38], Nepal (18.2%) [40] and Ethiopia (15.5%) [41]. Certain cultures may be responsible for different screening rates. Influenced by the traditional culture, pregnant women in China are inclined to hide their negative emotions. To maintain their personal and family image, they prefer to show their "positive" side to outsiders, while hiding the "negative" and "repressed" emotions inside. This Stoic character may have biased the screening score, resulting in a lower positive screening rate than that in other countries. However, this "suppressed" character can increase the possibility of unnoticed depressive symptoms and should be paid more attention.

Studies have found that socioeconomic factors are closely associated with the risk of depressive symptoms in pregnant women [24, 25, 30]. This is consistent with the findings of our study. However, some previous studies did not observe this association [26, 27]. This may be related to different economic conditions. A study in China showed a lower depressive symptom rate in pregnant women with an annual family income of more than 200,000 CNY compared to those with an income of less than 100,000 CNY [30]. A study in a southern state of India, which set the cut-off to 50,000 Indian Rupee(INR) and 1lakh INR did not find an association [27]. Concerning the economic level of Ningbo, we set a cutoff of 150,000 CNY (the average annual income of Ningbo households in the last 2 years). Our research showed a 1.762 time higher risk of depressive symptoms in pregnant women with an annual family income < 150,000 CNY than in those with ≥ 150,000 CNY. Pregnant women with low family incomes face more economic pressure during pregnancy and while raising babies. In the third trimester, pregnant women are more likely to have depressive symptoms for economic reasons as they face problems such as the imminent birth of their child and post-birth support. Family members should provide them with greater economic security. Additionally, the society and the government need to optimize policies to support them.

Previous studies have suggested that perinatal depressive symptoms are associated with poor sleep quality. A meta-analysis found that women who experienced poor sleep during pregnancy had a significantly higher risk of developing depressive symptoms(OR:3.72) [42]. For women in late pregnancy, due to frequent urination, fetal movement, leg cramps, and bad sleeping posture, sleep time is shortened, which may reduce sleep quality [43–45]. Although different methods are used to assess sleep quality, studies have shown that sleep quality is associated with depressive symptoms during late pregnancy [43, 46–48]. In our study, the risk of depressive symptoms in pregnant women with poor sleep quality was higher than that in those with good sleep quality (OR: 4.123, 95% CI: 2.764–6.163), which is consistent with previous studies. More attention should be given to sleep quality of pregnant women and appropriate guidance should be provided to those with poor sleep quality.

Ningbo is an open coastal city with a large fluid population. As many couples live separately, it is important to survey the influence of living separately ondepression among pregnant women. To the best of our knowledge, this is the first survey on this aspect. Our results showed that when couples did not live together, the risk of late pregnancy depression increased 2.054 times. The relationship between pregnant women and their partners is important for their emotional health [49, 50]. In recent years, under the influence of Western culture, the young Chinese population have abandoned the concept of a large family (living with their parents) and have become accustomed to living as couples. During pregnancy, especially in the late trimester, separation with partner may cause insecurity in pregnant women, increase their fear of childbirth, and cause depressive symptoms. We advocate that partnersprovide company to pregnant women during the perinatal period.

Alcohol consumption must be avoided during pregnancy. Fortunately, in this study, the drinking rate decreased from 38.71% before pregnancy to 1.34% during pregnancy, which is lower than that found by Amna [51]. However, we found that frequent drinking of partner increased the risk of late pregnancy depressive symptoms by 2.227 times. Partners’ alcohol consumption may cause tension and violence in relationships. Intimate partner violence is significantly associated with antenatal depressive symptoms [52, 53]. During the third trimester, the body size of pregnant women increases and they become less mobile. When faced with partner's drinking, the tension in the relationship is more likely to produce insecurity and depressive symptoms.

There are several limitations to this study. The major limitation of the study is that the findings are subject to reverse causation. For example it is possible that mothers experiencing prenatal depression are more likely to develop sleep disturbance. In the same way, fathers with depressed mothers may present with increased alcohol consumption than counterparts with non-depressed mothers. First of all, our hospital is the critical maternal rescue center in Ningbo, although our hospital only accounts for one-fifth of the city's births, the proportion of high-risk women is relatively large. That may biased the final results. Second, in this study, the dynamic psychological changes after delivery and the mental status of her babies were not analyzed, therefore a long-term prospective multi-center study is warranted. Therefore, our results should be interpreted with caution.

## Conclusion

This study found that the positive screening rate for depressive symptoms in late pregnancy was 9.9% at a tertiary hospital. Living away from partner, low annual family income, poor sleep quality, and partner’s frequent drinking habit were independent influencing factors for late pregnancy depressive symptoms. This study provides aninformation of higher depressive symptoms in pregnant women in the third trimester, which calls for a joint effort by society, healthcare professionals, families, and pregnant women. Since 2021, the Chinese Medical Association (CMA) has recommended the inclusion of depression screening for pregnant women into prenatal care. If the mother's score is considered positive, the obstetrician will recommend that they undergo a comprehensive clinical interview for further evaluation and appropriate treatment. Non-pharmacological and pharmacological therapies were used for the patients. And there were no information about mental health in prenatal classes for couples before 2022 in our prenatal care clinic. This year, we are offering this courses on mental health.

## Data Availability

The datasets used and/or analyzed during the current study are available from the corresponding author upon reasonable request.
